# Simultaneous Monitoring of Monoclonal Antibody Variants by Strong Cation-Exchange Chromatography Hyphenated to Mass Spectrometry to Assess Quality Attributes of Rituximab-Based Biotherapeutics

**DOI:** 10.3390/ijms22169072

**Published:** 2021-08-23

**Authors:** Fiammetta Di Marco, Thomas Berger, Wolfgang Esser-Skala, Erdmann Rapp, Christof Regl, Christian G. Huber

**Affiliations:** 1 Department of Biosciences, Bioanalytical Research Labs, University of Salzburg, Hellbrunner Straße 34, 5020 Salzburg, Austria; fiammetta.dimarco@sbg.ac.at (F.D.M.); Thomas.Berger@stud.sbg.ac.at (T.B.); wolfgang.esser-skala@sbg.ac.at (W.E.-S.); christof.regl@sbg.ac.at (C.R.); 2 Christian Doppler Laboratory for Innovative Tools for Biosimilar Characterization, University of Salzburg, Hellbrunner Straße 34, 5020 Salzburg, Austria; 3 Department of Biosciences, Computational Systems Biology Group, University of Salzburg, Hellbrunner Straße 34, 5020 Salzburg, Austria; 4 glyXera GmbH, Brenneckestraße 20—ZENIT, 39120 Magdeburg, Germany; e.rapp@glyxera.com; 5 Max Planck Institute for Dynamics of Complex Technical Systems, Sandtorstraße 1, 39106 Magdeburg, Germany

**Keywords:** monoclonal antibodies, biosimilar, MabThera^®^, Reditux™, post-translational modifications, strong cation-exchange chromatography, mass spectrometry, charged variant separation, pH gradient

## Abstract

Different manufacturing processes and storage conditions of biotherapeutics can lead to a significant variability in drug products arising from chemical and enzymatic post-translational modifications (PTMs), resulting in the co-existence of a plethora of proteoforms with different physicochemical properties. To unravel the heterogeneity of these proteoforms, novel approaches employing strong cation-exchange (SCX) high-performance liquid chromatography (HPLC) hyphenated to mass spectrometry (MS) using a pH gradient of volatile salts have been developed in recent years. Here, we apply an established SCX-HPLC-MS method to characterize and compare two rituximab-based biotherapeutics, the originator MabThera^®^ and its Indian copy product Reditux™. The study assessed molecular differences between the two drug products in terms of C-terminal lysine variants, glycosylation patterns, and other basic and acidic variants. Overall, MabThera^®^ and Reditux™ displayed differences at the molecular level. MabThera^®^ showed a higher degree of galactosylated and sialylated glycoforms, while Reditux™ showed increased levels of oligomannose and afucosylated glycoforms. Moreover, the two drug products showed differences in terms of basic variants such as C-terminal lysine and N-terminal truncation, present in Reditux™ but not in MabThera^®^. This study demonstrates the capability of this fast SCX-HPLC-MS approach to compare different drug products and simultaneously assess some of their quality attributes.

## 1. Introduction

In recent decades, monoclonal antibody (mAb)-based therapeutics have rapidly expanded in the biopharmaceutical industry [1]. mAbs find a multitude of clinical applications for the treatment of cancer, immunological disorders, and infections. Financial success and impeding patent expiry (or missing market launch in some countries) of many mAbs has spawned the market for biosimilars and copy products. The latter are generic drugs that do not fulfill regulatory criteria of biosimilarity but have been approved for therapeutic use independently. Demanding reduced pecuniary investment and temporal expenditure, biosimilars or copy products are highly attractive products for biopharmaceutical companies [2,3]. Rituximab, the first chimeric humanized mAb to be approved for the treatment of follicular lymphoma in 1997, was succeeded by numerous biosimilars and copy products that were developed all around the world [4], such as Reditux™ in India [5] or Rixathon in the European Union [6].

In order to gain approval for clinical use as a biosimilar, biosimilarity to the originator product must be proven rigorously by analytical means, as different manufacturing processes may lead to variability of biotherapeutics. Protein heterogeneity arises from a variety of post-translational modifications (PTMs), leading to the co-existence of numerous proteoforms with different physicochemical properties [7]. Thus, characterization of mAb modifications is a prerogative to prove safety and efficacy of a biosimilar [8,9,10,11]. 

Among the plethora of PTMs, which mAbs may undergo during manufacturing or storage, are N-terminal pyroglutamate formation, C-terminal enzymatic lysine clipping, enzymatic asparagine glycosylation, chemical lysine ε-amino group glycation, and asparagine deamidation, resulting in the formation of acidic or basic variants. Moreover, depending on the site affected by the PTM, conformational changes can be induced in the protein, leading to a difference in the mAb surface charge distribution and/or tertiary structure [12,13].

As PTMs directly and interdependently affect the charge of the protein, capillary electrophoresis [14] and ion-exchange (IEX) high-performance liquid chromatography (HPLC) are used as gold standard techniques to assess the quality of proteins and to allow for separate control of individual proteoforms [15,16,17]. Conventional IEX approaches comprise the use of non-volatile salt gradients, where ionic strength is increased during the chromatographic run. This allows for separation of charge variants according to surface charge at intact proteoform level [17]. An alternative approach employs a pH gradient using volatile buffers (e.g., formic acid/formate, acetic acid/acetate, ammonium/ammonia), eluting the different proteoforms depending on their isoelectric point (pI), hence their acidic properties [16,18,19,20]. In contrast to the conventional non-volatile salt gradient, a pH gradient involving volatile mobile phases facilitates direct hyphenation to mass spectrometry. 

In the last few years, hyphenation of weak or strong cation-exchange HPLC with mass spectrometry (WCX-HPLC-MS or SCX-HPLC-MS) was applied for characterization of intact mAb charge variants [21,22,23,24,25,26,27]. Füssl et al. optimized charge variant separation by SCX-HPLC-MS for a variety of mAbs, including rituximab [24], but they showed and discussed mass spectra only for adalimumab [23,26], trastuzumab [23,26], bevacizumab [23], and cetuximab [25]. The combination of separation and mass detection allows proteoform discrimination based on acidic properties and mass, adding a new dimension to variant characterization. For instance, asparagine deamidation causes a mass shift of ≈1 Da, which is impossible to resolve by MS at intact mAb level. However, the conversion of an amide to a carboxylic functional group leads to the formation of acidic variants partially separable by SCX-HPLC [24]. On the contrary, the majority of mAb glycoforms cannot be separated by SCX but may be discriminated according to their different masses. Here, we employ the SCX-HPLC-MS approach [24] to characterize intact proteoforms of rituximab biotherapeutics, aiming to assess some aspects of biosimilarity between the drug product MabThera^®^ and its Indian copy product Reditux™. Using this approach, molecular differences between the two drug products can be highlighted at intact protein level with minimal sample preparation and applying just a fifteen-minute-gradient separation. Two studies demonstrated that the two drugs showed no clinical differences in terms of toxicity, tumor response rates, prolonged progression-free survival, and overall survival when prescribed for diffuse large B-cell lymphoma [28,29]. However, from a molecular perspective, MabThera^®^ and Reditux™ exhibited a significant variability [14,30,31,32,33]. Nupur et al. [33] used a multi-analytical approach to assess the biosimilarity of the two biotherapeutics and reported an increased level of core-afucosylated and oligomannose glycans in Reditux™ compared to MabThera^®^, as well as differences in charge variant patterns.

Unlike previous studies of biosimilarity assessment of the two drug products [14,30,31,32,33], our study comprises a novel analytical approach where charge variants of MabThera^®^ and Reditux™ are separated under native conditions using volatile salts, allowing the hyphenation of SCX-HPLC to MS and leading to better spatially resolved mass spectra of rituximab proteoforms, as explained in detail in Reference [34]. Thereby, differences between glycosylation patterns of MabThera^®^ and Reditux™ should become observable, also in terms of low-abundant glycoforms. Moreover, we assess the feasibility of separating deamidated and glycated proteoforms from the main variants. The suitability of the method to detect these modifications is investigated by inducing forced deamidation and glycation on rituximab followed by corroboration of PTM hotspots via peptide mapping. Additionally, carboxypeptidase B digestion is performed at intact protein level to confirm the presence of abundant C-terminal lysine variants in Reditux™. Collecting all molecular data, we aim at assessing the range of variability of the two biotherapeutics in terms of several quality attributes including glycosylation and C-terminal lysine variants. Finally, we demonstrate that the method is suitable to assess aspects of biosimilarity for different rituximab biosimilars and copy products. 

## 2. Results

### 2.1. SCX-HPLC-MS Charge Patterns of MabThera^®^ and Reditux™ 

**Figure 1:**
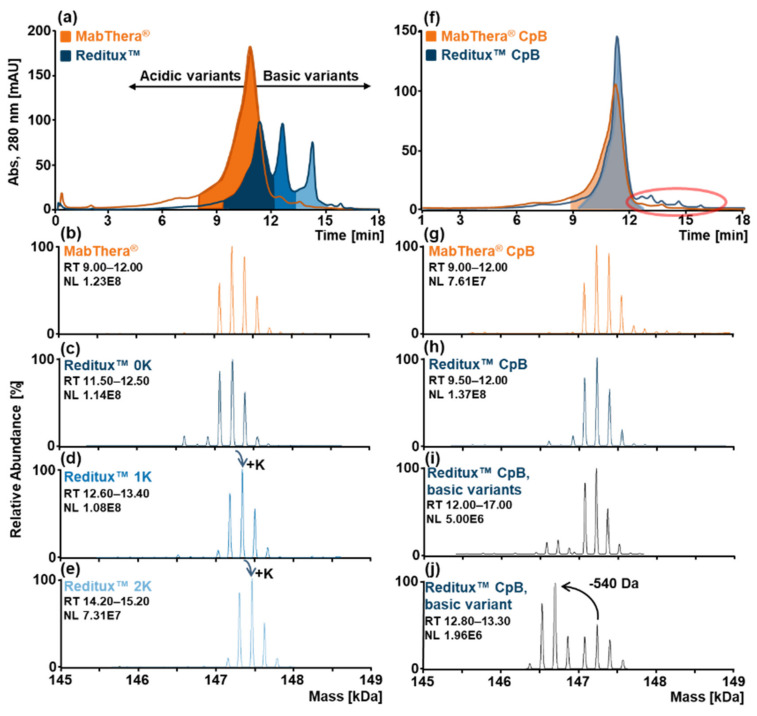
SCX-HPLC-MS analyses of MabThera^®^ (orange) and Reditux™ (blue). (**a**) SCX-HPLC-MS chromatogram of intact MabThera^®^ (orange) and Reditux™ (blue). Deconvoluted mass spectrum of (**b**) MabThera^®^ main variant without C-terminal lysine (0K), (**c**) Reditux™ variant without C-terminal lysine (0K), (**d**) Reditux™ variant with one C-terminal lysine (1K), (**e**) Reditux™ variant with two C-terminal lysine (2K). The three deconvoluted spectra of Reditux™ exhibit the same pattern shifted by +128 Da, corresponding to a lysine residue. (**f**) SCX-HPLC-MS chromatogram of MabThera^®^ (orange) and Reditux™ (blue) after treatment with carboxypeptidase B (CpB) to remove the C-terminal lysine. The red circle indicates basic variants that are present in Reditux™ and almost completely absent in MabThera^®^. Deconvoluted mass spectrum of (**g**) MabThera^®^ main variant with complete clipping of C-terminal lysine (0K), (**h**) Reditux™ variant with complete clipping of C-terminal lysine (0K), (**i**) Reditux™ basic variants eluting at RT 12.00–17.00 min, (**j**) Reditux™ basic variant eluting at RT 12.80–13.30 min. Annotations of the spectra are reported in Tables S2–S6.

Rituximab is a mAb exhibiting an average basic pI of 9.4 [18]. In order to separate charge variants of the originator MabThera^®^ and its Indian copy product Reditux™, an SCX-HPLC-MS approach involving an increasing pH gradient of pH ≈ 8.9–10.2 was applied over 15 min. In this fashion, the acidic proteoforms (deamidated, glycated, or sialylated variants) elute first while the basic proteoforms (C-terminal lysine variants) elute later with respect to the main variant. The patterns of charge variants of the two drug products obtained were then compared based on the retention time of the eluting proteoform and their associated mass spectra (Figure 1). The chromatogram of MabThera^®^ showed a prominent peak (orange, Figure 1a) due to the elution of the main proteoforms as confirmed by the associated deconvoluted mass spectra (Figure 1b). On the other hand, for Reditux™, the pattern of charge variants is characterized by three main chromatographic peaks (blue, Figure 1a). The three deconvoluted mass spectra of Reditux™ showed similar glycosylation patterns, exhibiting a mass shift of 128 Da between the different chromatographic peaks (Figure 1c–e). This mass shift, together with the retention time of the variants confirmed a basic nature of the modification and suggested the presence of C-terminal lysine variants in Reditux™ in higher abundance as compared to MabThera^®^.

To further confirm this hypothesis, the two biotherapeutics were digested with carboxypeptidase B (CpB) to enzymatically remove C-terminal lysine(s), and the treated samples were re-analyzed by SCX-HPLC-MS. After CpB treatment, the chromatogram of Reditux™ displayed one main peak, confirming the removal of the C-terminal lysine (Figure 1f). The deconvoluted spectra associated with the main peaks of the two drugs further support this interpretation (Figure 1g,h).

Interestingly, even after CpB treatment, the presence of other basic variants was noted in Reditux™ (red circle, Figure 1f) but not in MabThera^®^. The deconvoluted mass spectrum of the entire range of the basic variants (Figure 1i) showed that most of the basic proteoforms exhibited similar (or non-resolvable) masses of the variants. However, a low-abundance basic variant showed a preserved glycosylation pattern shifted by −540 Da (Figure 1j). This variant eluted in a retention time range of 12.80–13.30 min and, based on orthogonal mAb subunit data (see Figure S1), probably arose from the truncation of the N-terminal peptide (pyroGlu1-Ser5) of the light chain.

### 2.2. Forced Deamidation of MabThera^®^ and Reditux™

**Figure 2:**
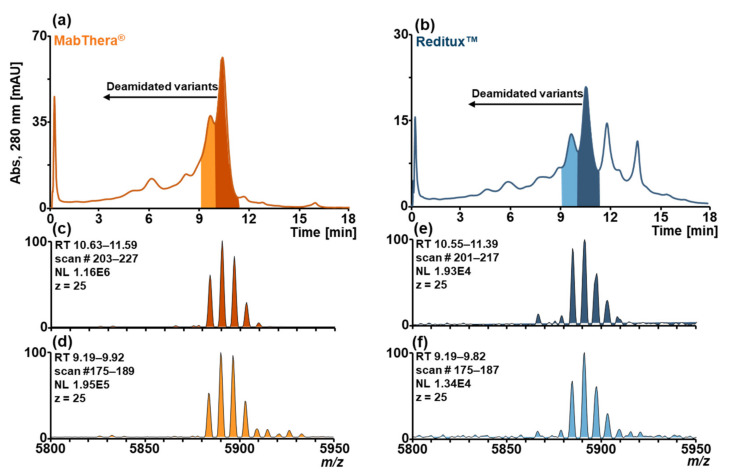
SCX-HPLC-MS chromatograms of forced deamidated (**a**) MabThera^®^ (orange) and (**b**) Reditux™ (blue). Additional chromatographic peaks due to deamidated variants are indicated. Magnification of charge state 25+ of the mass spectrum associated with (**c**) the peak at RT 10.63–11.59 min of MabThera^®^, (**d**) the peak at RT 9.19–9.92 min of MabThera^®^, the peak at RT 10.55–11.39 min of Reditux™, and (**f**) the peak at RT 9.19–9.82 min of Reditux™. No clear differences can be observed between the spectra (**c**,**d**) and (**e**,**f**) due to the inability of MS to resolve the mass shift of deamidation (≈1 Da) at the intact level.

To investigate the suitability of SCX-HPLC-MS to separate deamidated proteoforms, MabThera^®^ and Reditux™ samples were stressed with a basic solution of ammonium bicarbonate at 37 °C over 3 days and analyzed by SCX-HPLC-MS. Chromatograms of MabThera^®^ (Figure 2a) and Reditux™ (Figure 2b) after forced deamidation displayed additional peaks at lower retention time (lower pH of the mobile phase) indicating the presence of acidic variants. Comparison of the spectra of the main peak and the adjacent left one of MabThera^®^ (Figure 2c,d) and Reditux™ (Figure 2e,f) to each other did not reveal a clear difference in mass, confirming the existence of an acidic variant not resolvable by MS.

Peptide mapping was performed on non-stressed and forced deamidated samples to confirm the presence of deamidation and unravel the modification hotspots (Figure S2). Stressed samples resulted in a higher deamidation degree compared to the non-stressed samples. In particular, sites N319, N388, N393, and N394 of the heavy chain (HC) exhibited a higher amount of degradation. These results are consistent with previously published data attributing the main region affected by deamidation in the “PENNY” peptide (SNQPENNYK) [35,36]. Moreover, sites N425 and N438 of the HC and sites N136, N137, N151, N157 of the light chain (LC) were deamidated practically to the same extent in non-stressed and stressed samples. This could explain why the main peak in the chromatograms of untreated MabThera^®^ and Reditux™ (Figure 1a) displayed a preceding shoulder. As acidic variants, deamidated proteoforms would elute earlier, together with other acidic variants carrying mainly sialylation and glycation compared to the main variant. 

### 2.3. Forced Glycation of MabThera^®^ and Reditux™

**Figure 3:**
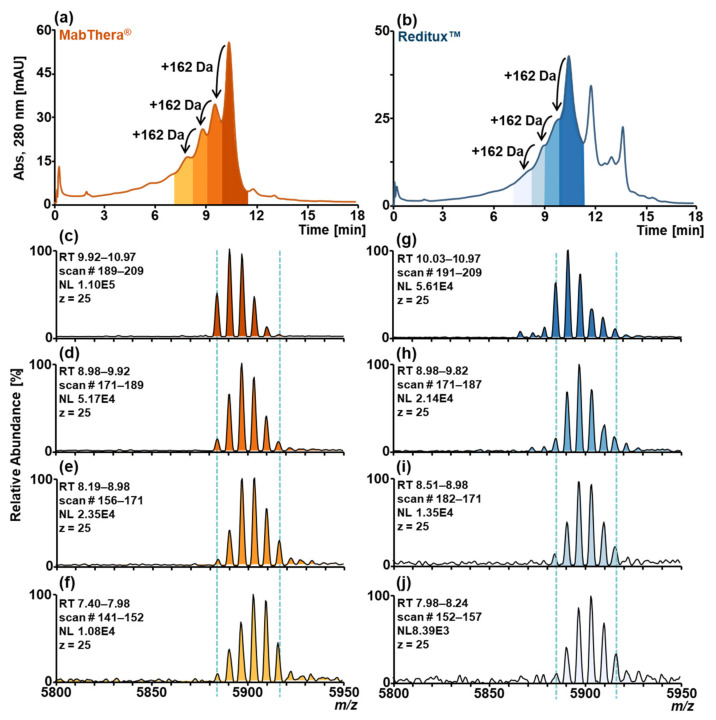
SCX-HPLC-MS chromatograms of forced glycated (**a**) MabThera^®^ (orange) and (**b**) Reditux™ (blue). Additional chromatographic peaks due to glycated variants are indicated by arrows (+162 Da). Magnification of charge 25+ of mass spectrum associated with (**c**) peak at RT 9.83–10.97 min of MabThera^®^, (**d**) peak at RT 8.98–9.92 min of MabThera^®^, (**e**) peak at RT 8.19–8.98 min of MabThera^®^, (**f**) peak at RT 7.40–7-98 min of MabThera^®^, (**g**) peak at RT 10.03–10.97 min of Reditux™, (**h**) peak at RT 8.98–9.82 min of Reditux™, (**i**) peak at RT 8.51–8.98 min of Reditux™, (**j**) peak at RT 7.98–8.24 min of Reditux™. A shift of 162 Da in the glycosylation pattern due to the glycation bias can be observed going to the more acidic variants (spectra c–f and g–l).

To assess the capability of the SCX-HPLC-MS method to separate glycated variants, glycation was induced in samples of MabThera^®^ and Reditux™ using a 1.0 mol·L^−1^ glucose solution, and the stressed samples were then analyzed by SCX-HPLC-MS (Figure 3a,b). The chromatograms of both MabThera^®^ (Figure 3a) and Reditux™ (Figure 3b) displayed additional peaks due to acidic variants (black arrow, Figure 3a,b) compared to the non-stressed samples (Figure 1a). Due to the isobaricity of glucose with galactose (monosaccharides present in glycans) it is impossible to distinguish at the intact level between increased galactosylation or glycation based on the mass shift (+162 Da in both cases). However, SCX-HPLC separation adds a new dimension to the detection of this modification. Using SCX-HPLC-MS, the glycated variants showed partial chromatographic resolution, and the mass spectra of the differently glycated proteoforms can be observed separately, showing a shift towards heavier glycoforms trending towards more acidic variants (mass spectra Figure 3c–f and g–j).

As an orthogonal method, peptide mapping was performed in order to characterize glycation hotspots (Figure S3). Both stressed MabThera^®^ and Reditux™ exhibited a higher degree of glycation compared to the non-stressed samples. The same 19 lysine residues were glycated in both drugs to a different extent. In particular, the lysine residues with the highest glycation degree in both stressed and non-stressed samples were K151 for the HC and K182, 187, and 189 for the LC (Figure S3).

### 2.4. Glycosylation Patterns of MabThera^®^ and Reditux™

The elucidation of the glycosylation pattern in mAbs is fundamental to assess the biosimilarity of two drug products because different glycoforms may have different physicochemical properties and thus affect the safety and efficacy of the biotherapeutics [8,10]. The MS coupled to SCX using a pH gradient of volatile salts allows the acquisition of mass spectra of glycoforms under native conditions (Figure S4). The mass spectra of MabThera^®^ and Reditux™, untreated and after CpB treatment, were deconvoluted to obtain glycoform masses (Figure 1b–e,g,h). To elucidate glycan structures, *N*-glycans were released by PNGase F treatment and labeled with aminopyrene-1,3,6-trisulfonic acid, and analyzed by multiplexed capillary gel electrophoresis with laser-induced fluorescent detection (Table S1). Glycoforms at the intact level were characterized and annotated (Tables S2–S6). Masses of major variants could be assessed with an accuracy below 20 ppm, while the masses of the minor variants were assessed with up to 50 ppm mass error.

**Figure 4:**
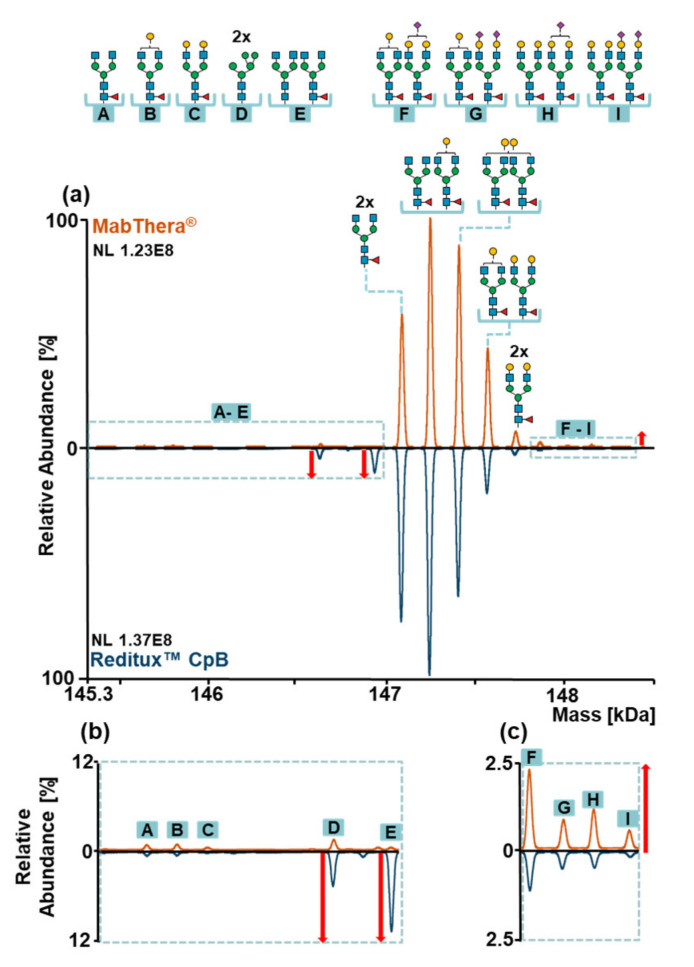
(**a**) Mirror plot of the deconvoluted mass spectra of MabThera^®^ (orange) and Reditux™ (blue) after carboxypeptidase B treatment (CpB). Glycan structures of main glycoforms are reported above the peaks, while minor glycoforms are indicated with the letters A–I. Differences in relative abundances of glycoforms can be observed between MabThera^®^ and Reditux™. (**b**) Magnification of minor glycoforms A–E. Red arrows indicate an increased amount of oligomannose (D) and afucosylated (E) glycoforms in Reditux™ compared to MabThera^®^. (**c**) Magnification of minor glycoforms F–I. The red arrow indicates an increased amount of sialylated glycoforms (F–I) in MabThera^®^ compared to Reditux™. Annotations of the spectra are reported in Tables S2 and S5. Glycan structures, names, and compositions are collected in Table S1.

MabThera^®^ and Reditux™ after CpB treatment (Figure 4) exhibited the same glycoforms but differences in their relative abundances. For both drugs, the most abundant glycoform was A2G0F/A2G1F. However, MabThera^®^ showed a higher galactosylation degree of the main glycoforms compared to Reditux™, indicated by the highest abundance of glycoform A2G1F/A2G1F (or A2G0F/A2G2F) compared to A2G0F/A2G0F (Figure 4a). Concerning minor variants, the copy product showed a higher degree of oligomannose and afucosylated variants (variants D –E, Figure 4b) and a lower abundance of sialylated variants (variants F –I, Figure 4c). 

**Figure 5:**
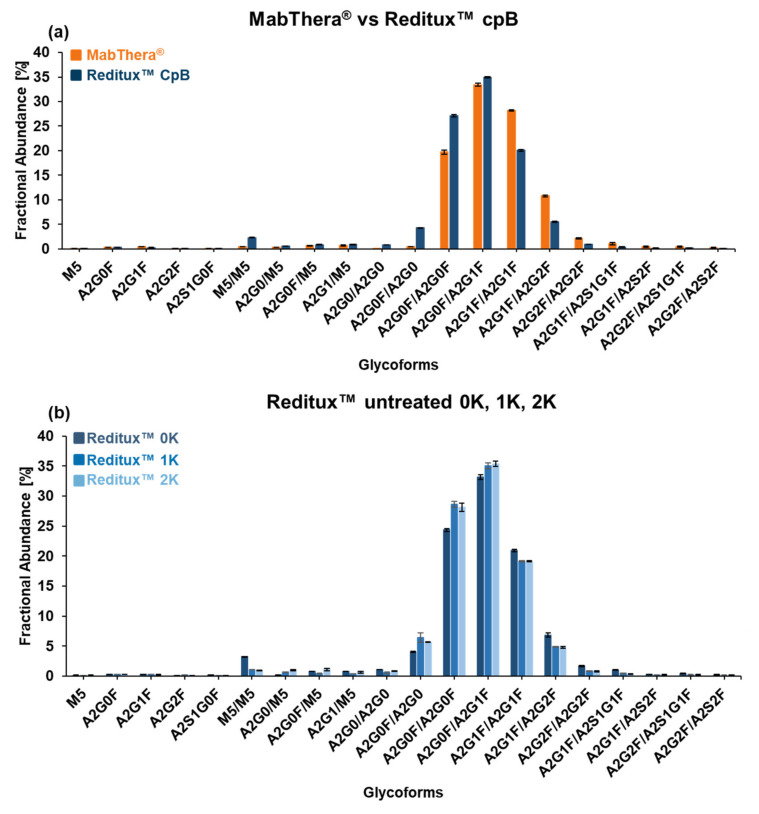
Glycoform fractional abundance was obtained by area integration of extracted ion current chromatograms (EICCs). Bar charts of glycoform fractional abundance of (**a**) MabThera^®^ and Reditux™ after carboxypeptidase B (CpB) tretament, (**b**) Reditux™ different lysine variants (0K, 1K, 2K). Columns represent averages and error bars, and the 95% confidence interval was calculated using a t-test from triplicate analysis. Glycoform name is reported in the x-axis. Table S7 reports the results in percentage. Glycan structures, names, and compositions are collected in Table S1.

In order to semi-quantify these differences, our in-house software tool fragquaxi was utilized. The script is able to obtain fractional abundances of glycoforms from mass spectrometric data by quantification via extracted ion current chromatogram (EICC) integration. The results (Figure 5a and Table S7) confirmed what we already observed in the deconvoluted mass spectra. For instance, the galactosylation level for the main glycoforms was 32.67 ± 0.05% for MabThera^®^ and 23.86 ± 0.18% for Reditux™ at 95% confidence level. The fractional abundances of glycoform M5/M5 were 0.45 ± 0.02% and 2.30 ± 0.03%, and the one of the afucosylated glycoform A2G0F/A2G0 was 0.44 ± 0.01% and 4.28 ± 0.05% for MabThera^®^ and Reditux™ at 95% confidence level, respectively. Moreover, the fractional abundances of sialylated proteoforms were 2.24 ± 0.47% for the originator and 0.79 ± 0.10% for the copy product at 95% confidence level. The semi-quantitative glycoform assessment at intact level was supported by glycopeptide analyses (see Figure S5).

Using the same approach with fragquaxi, glycosylation patterns of Reditux™ representing different lysine variants (0K, 1K, 2K) were compared (Figure 5b). Interestingly, differences were observed between the 0K variant and the 1K or 2K variants. The 0K variant showed a higher abundance of variant M5/M5 (3.24% ± 0.05% 0K, 1.07% ± 0.03% 1K, 0.93% ± 0.03% 2K at 95% confidence level) and a slightly higher degree of galactosylation (25.52% ± 0.37% 0K, 22.85% ± 0.10% 1K, 22.77% ± 0.14% 2K at 95% confidence level) compared to the 1K or 2K variants, which do not exhibit substantial differences.

**Figure 6:**
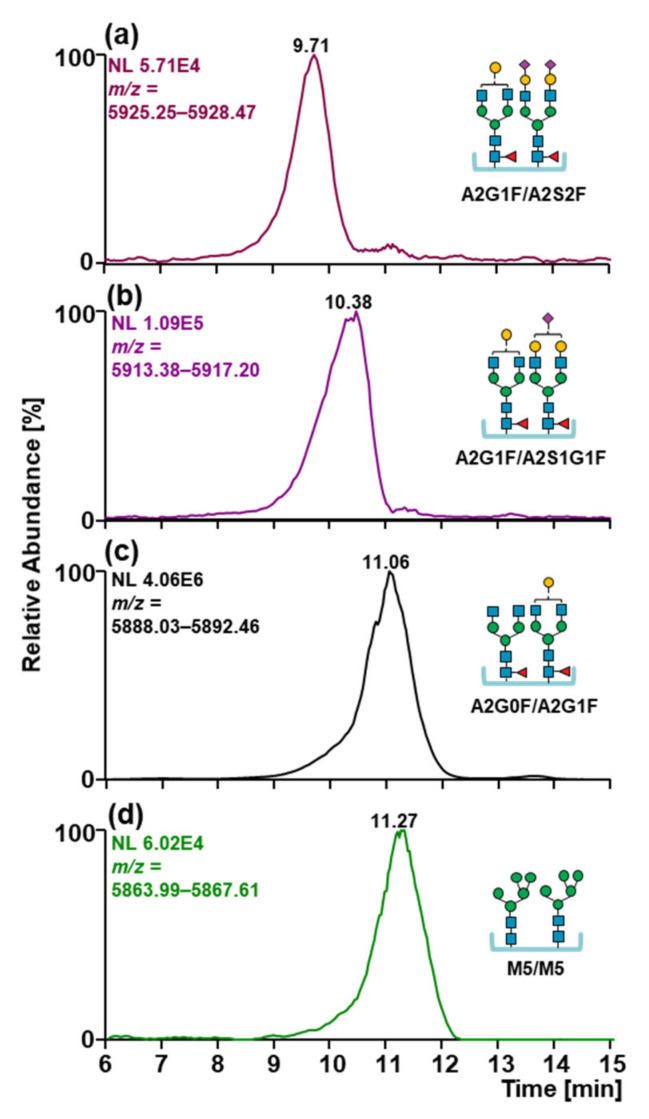
Extracted ion current chromatograms of the most abundant charge state of the glycoform (**a**) A2G1F/A2S2F, (**b)** A2G1F/A2S1G1F, (**c**) A2G0F/A2G1F, and (**d**) M5/M5. Glycoform carrying sialic acids eluted at lower RT while oligomannose type to higher RT. Glycan structures, names, and compositions are collected in Table S1.

Other than mass detection, SCX-HPLC-MS is also able to partially separate sialylated glycoforms and oligomannose type glycoforms from the main glycoforms. In Figure 6, EICCs of the main charge state of monosialylated, disialylated, and oligomannose glycoform of MabThera^®^ are reported. The presence of sialic acids in the glycan structures caused a shift towards lower retention times (from 11.06 to 10.38 to 9.71 min) due to the acidic nature of this modification causing an additional negative charge. Surprisingly, oligomannose type glycoforms can be partially resolved and eluted at higher retention time (from 11.06 to 11.27 min). On the contrary, a different galactosylation degree (from 0 to 4 galactoses) on the biantennary glycans did not affect the separation of the glycoforms.

## 3. Discussion

In this study, two rituximab-based biotherapeutics, the originator MabThera^®^ and its copy product Reditux™, were characterized by SCX-HPLC-MS in order to assess some of their molecular quality attributes. The use of a pH gradient allowed to separate proteoforms based on their pI value, hence their acidic properties. Moreover, the use of volatile salts enabled the hyphenation with MS. In this fashion, an additional dimension to conventional SCX-HPLC-UV was added, and proteoforms were characterized based on their retention properties (acidic or basic variants) but also their masses. 

Reditux™ showed that C-terminal lysine variants were not present in MabThera^®^ (Figure 1a). It is generally accepted that C-terminal lysines do not significantly affect antibody function, because C-terminal lysines are cleared when entering circulation as well as due to distal positioning of the PTM from the antigen-binding domain [37,38]. However, one study demonstrated that the presence of C-terminal lysine can negatively affect complement-dependent cytotoxicity (CDC), decelerating the process of hexamerization at the cell surface [39]. 

After CpB digestion, basic variants can be observed in the chromatogram of Reditux™ (Figure 1f). Most of these variants exhibited masses indistinguishable from that of the main glycoforms of Reditux™; hence, they carried some modifications with a basic nature not resolvable by MS at the intact protein level [40], e.g., C-terminal proline amidation, succinimide formation, methionine oxidation, glutamic acid to pyroglutamate conversion, aspartate isomerization, or disulfide shuffling [41,42]. However, one variant displayed a difference in mass of −540 Da and a preserved glycosylation pattern. We, therefore, postulate this variant to arise from N-terminal cleavage of the first five residues in the light chain (Figure S1). It is known that N-terminal truncation can occur due to an erroneous recognition of the signal peptide cutting point, leading to a shift in the cleavage site [12,43]. N-terminal heterogeneity can affect mAb bioactivity because of its location close to the complementary determining region. The presence of this modification in Reditux™ but not in MabThera^®^ could be a critical quality attribute in assessing their biosimilarity, but requires additional confirmation via bioassay.

Acidic variants were partially resolved using this method. No clear differences were observed between Reditux™ and MabThera^®^, both showing a low abundance of deamidated and glycated proteoforms (Figure 1a,f). Unfortunately, this highlighted the limitation of this technique for the characterization of these PTMs: a low deamidation level can only be observed as a shoulder of the main peak and cannot be quantified via EICC integration because of the inability of MS to discriminate this small mass shift at intact mAb level (Δm ≈ +1 Da). Because of the isobaricity with additional galactose in the glycans, the quantitation via EICC is also impossible for glycation, but the identification of this PTM by SCX-HPLC-MS can be based on the presence of a left shoulder in the main peak and a shift of +162 Da in the glycosylation pattern, making it distinguishable from deamidation. However, we demonstrated the ability of this method to detect this PTM when the level of the modification is higher (Figure 2 and Figure 3). This was performed upon inducing the modification in the samples with stressing protocols. In this way, we mimicked storage conditions enhancing the formation of such artefacts in the biotherapeutics that cause an increased level of deamidation and glycation on the drug substance but also, in the case of glycation, to mimic a high concentration of glucose in the culture medium during the fermentation process [44,45].

Glycosylation can affect the mAb structure and activity, in particular when the mechanism of action involves antibody-dependent cellular cytotoxicity (ADCC) and CDC [46,47]. Specifically, a higher degree of galactosylation increases CDC activity, enhancing the binding with the protein complement component 1q (C1q) [48]. Furthermore, oligomannose type and core-afucosylation increase ADCC activity, strengthening the FcγIIIa receptor binding affinity [49,50]. On the other hand, a higher extent of sialylation negatively affects ADCC [51]. Reditux™ showed a higher degree of oligomannose, afucosylated, and asialylated variants and thus a possibly higher ADCC activity, while MabThera^®^ exhibited a higher degree of galactosylation, leading to a possibly increased CDC activity (Figure 4 and Figure 5). Interestingly, Reditux™ lysine variants showed significant differences in glycosylation patterns (Figure 5). The correlation between C-terminal modifications and glycosylation is still not clear and it has only been observed in few studies [52].

In conclusion, SCX-HPLC-MS analysis permitted the characterization of several PTMs occurring in MabThera^®^ and Reditux™. The results revealed structural differences for both drugs in terms of C- and N-terminal variants and glycosylation. We hypothesize that this heterogeneity arises from the use of different expression host cells and/or different culture conditions (temperature, pH, CO_2_ concentration, time, bioreactor size, etc.) in the bioprocesses that may tune the glycosylation profile and the C-terminal lysine variants of these biotherapeutics [53,54,55]. However, despite the molecular heterogeneity of the proteoforms observed in the two biotherapeutics, no clinical differences between the two drugs in terms of toxicity were observed when prescribed for the treatment of diffuse large B-cell lymphoma [28,29].

The study reported an example of the heterogeneity that can be characterized in biotherapeutics using SCX-HPLC-MS. The structural characterization of mAbs is of crucial importance not only to assess some aspects of biosimilarity between different drug products, but also to potentially find application in batch-to-batch comparison in the future. This work constitutes the basis for simultaneously monitoring multiple attributes in rituximab-based biotherapeutics using a fast SCX-HPLC-MS method.

## 4. Materials and Methods

### 4.1. Materials

The Rituximab-based biopharmaceuticals used were the commercially available MabThera^®^ (Lot N7042, exp. May 2017, 10 mg mL^−1^, F. Hoffmann-La Roche Ltd., Basel, Switzerland) and its Indian copy product Reditux™ (Lot RIAV02616, exp. October 2018, 10 mg mL^−1^, Dr Reddys Laboratories Ltd., Hyderabad, India) and purchased from a local pharmacy store. Acetic acid, ammonium bicarbonate, ammonium hydroxide, D(+)-Glucose, guanidine hydrochloride, tris(2-carboxyethyl)phosphine, and iodoacetamide were purchased from Sigma-Aldrich (St. Louis, MO, USA). Formic Acid was obtained from Fluka Analytical (Steinheim, Germany), and Acetonitrile and ammonium acetate were provided by VWR chemicals (Radnor, PA, USA). Water was purified in-house by a Milli Q Integral 3 system (Merck Millipore (Burlington, MA, USA). Trypsin and chymotrypsin, MS grade, were purchased from Promega (Fitchburg, WI, USA) and recombinant Carboxypeptidase B from Roche Diagnostics GmbH (Mannheim, Germany). Amicon Ultra Centrifugal Filter Device 10 kDa and 3 kDa cutoff, cellulose membrane, were all purchased from Sigma-Aldrich (St. Louis, MO, USA). Sartorius Vivaspin 30 kDa cutoff centrifugal filters were obtained from Sartorius (Göttingen, Germany).

### 4.2. Sample Preparation

For SCX-HPLC-MS analyses of intact antibodies, MabThera^®^ and Reditux™ were buffer-exchanged in 25 mmol·L^−1^ ammonium bicarbonate and 30 mmol·L^−1^ acetic acid aqueous solution (mobile phase A) using the 30 kDa cutoff Vivaspin filters three times with a final volume of 500 µL and a sample load of 100 µL.

Removal of heavy-chain C-terminal lysine in both MabThera^®^ and Reditux™ was performed using recombinant carboxypeptidase B. Two mg of antibody were buffer-exchanged in 150 mmol·L^−1^ ammonium acetate using the 30 kDa cutoff Vivaspin filters. Subsequently, 0.40 mg of carboxypeptidase B were added to achieve a substrate-to-enzyme ratio of 5:1, and the mixture was incubated for 30 min at room temperature while shaking. Prior to SCX-HPLC-MS analyses, the digested antibody was buffer exchanged in mobile phase A using the 30 kDa cutoff Vivaspin filters.

Forced deamidation of both MabThera^®^ and Reditux™ was carried out with a basic solution of 200 mmol·L^−1^ ammonium bicarbonate, pH 8.2. The antibody was buffer exchanged in 200 mmol·L^−1^ ammonium bicarbonate using the Amicon 10 kDa cutoff filters. The samples were then incubated for 72 h at 37 °C in a thermoshaker at 500 rpm. Finally, prior to SCX-HPLC-MS analysis, the samples were buffer-exchanged in mobile phase A using the Amicon 10 kDa cutoff filters.

Forced glycation of both MabThera^®^ and Reditux™ was performed by adding 200 µL of a 1.0 mol·L^−1^ glucose solution to 2.0 mg of antibody in 200 µL for a final concentration of 500 mmol·L^−1^ glucose. Afterwards, the samples were incubated for 24 h at 40 °C in the dark in a thermoshaker at 500 rpm. Prior to SCX-HPLC-MS analyses, the samples were buffer exchanged in mobile phase A using the Amicon 10 kDa cutoff filters.

A peptide digest of untreated and stressed MabThera^®^ and Reditux™ was prepared to identify glycation and deamidation hotspots. At first, 5.0 µg of antibody for each sample were diluted in 50 µL of H_2_O. Subsequently, for denaturation and reduction of disulfide bridges, a final concentration of 3.0 mol·L^−1^ of guanidine hydrochloride and 5.0 mmol·L^−1^ of tris(2-carboxyethyl)phosphine were added in a total volume of 110 µL. Samples were left for 1.0 h at 50 °C under shaking. Alkylation was then carried out in 20 mmol·L^−1^ iodoacetamide for 1.0 h at 22 °C in the dark while shaking. Prior to digestion, alkylated antibody was buffer-exchanged into 150 mmol·L^−1^ ammonium acetate using the Amicon 10 kDa cutoff filters to a final volume of 50 µL. Afterward, untreated and deamidated MabThera^®^ and Reditux™ samples were digested using 0.50 µg of trypsin (substrate-enzyme ratio 10:1), while untreated and glycated MabThera^®^ and Reditux™ were digested using 0.50 µg of trypsin and 0.50 µg of chymotrypsin (substrate-enzyme ratio 10:1). The digestion was left overnight at 37 °C while shaking.

For xCGE-LIF-based analysis, released N-glycans were prepared using a glyXprep™ kit (glyXera, Magdeburg, Germany). Following the kit instruction guide, 10 µg of each mAb (MabThera^®^ and Reditux™) dissolved in 6.0 µL ultrapure water were supplemented with 1.0 µL Denaturation Solution (kit) and 1.0 µL 1.0 mol·L^−1^ DTT_aq_. For protein denaturation and linearization, the mixture was incubated for 10 min at 60 °C. Additives were neutralized by addition of 2.0 µL Neutralization Solution (kit). Glycans were released by addition of 1.0 µL PNGase F Solution (kit) and incubation for 30 min at 37 °C. After glycan release, samples were dried in a vacuum concentrator and labeled with the fluorescence dye 8-aminopyrene-1,3,6-trisulfonic acid (APTS). Therefore, samples were resolved in 2.0 µL ultrapure water, 2.0 µL APTS Labeling Solution (kit), and 2.0 µL ReduX Solution (kit); mixed carefully; and incubated for 3.0 h at 37 °C. The labeling reaction was stopped by adding 100 µL Stopping Solution (kit). According to the kit instruction guide, post derivatization cleanup was performed by HILIC-SPE as follows: samples were applied to a filter plate well containing 200 µL glyXbead Slurry (kit) and incubated for 5.0 min at ambient temperature for binding, followed by washing and elution steps.

### 4.3. SCX-HPLC-MS Analyses of MabThera^®^ and Reditux™

SCX-HPLC-MS analyses of untreated, digested, and stressed MabThera^®^ and Reditux™ were carried out on an HPLC system (Ultimate 3000 UHPLC, Thermo Fisher Scientific^TM^, Germering, Germany) hyphenated to a quadrupole-OrbitrapTM mass spectrometer (Q Exactive™ Plus Hybrid Quadrupole-Orbitrap™ mass spectrometer, Thermo Fisher Scientific, Bremen, Germany). Separation was performed on a MAbPac SCX-10 RS 5 µm, 2.1 × 50 mm column (Thermo Fisher Scientific, Sunnyvale, CA, USA) using a mobile phase A comprising 25 mmol·L^−1^ ammonium bicarbonate and 30 mmol·L^−1^ acetic acid in water (pH 5.3) and a mobile phase B comprising 10 mmol·L^−1^ ammonium hydroxide in water (pH 10.8). A pH gradient was applied consisting of 75 to 99% mobile-phase B in 15 min followed by a flushing step with 99% B for 5 min and an equilibration step with 10% B for 10 min and 75% B for 25 min, flowrate 400 µL min^−1^, T 25 °C, and UV detection at 280 nm. 100 µg of antibody were injected per run. The Q Exactive™ Plus mass spectrometer was equipped with the BioPharma Option, which enables operation in high-mass range mode (HMR) with detection up to *m/z* 8000. The source parameters were set as follows: spray voltage 3.6 kV, capillary temperature 275 °C, sheath gas 20 (arbitrary units), auxiliary gas 5 (arbitrary units), probe heater temperature 275 °C. MS parameters were in-source CID 100, S-lens RF level 200, polarity positive, trapping gas pressure setting 1, AGC target 3e6, maximum IT 200 ms, *m/z* range 2500–8000, microscans 10, and a resolution setting of 17,500 (@ *m/z* 200).

### 4.4. Peptide Mapping of Untreated and Stressed MabThera^®^ and Reditux™ by Nano RP-HPLC-MS/MS

Peptide mapping measurements were carried out on a Thermo Scientific™ Ultimate 3000 RSLCnano UHPLC (Thermo Fisher Scientific, Germering, Germany) hyphenated to a Q Exactive™ Plus Hybrid Quadrupole-Orbitrap™ mass spectrometer. Injection volume was 1 µL. Chromatographic separation was achieved using a 200 cm micro pillar array column, µPAC™ (PharmaFluidics, Ghent, Belgium) operated at 50 °C. Mobile phase A was composed of water with 0.1% formic acid and a mobile phase B composed of acetonitrile with 0.1% formic acid. To accelerate column equilibration, flowrate was at first set at 750 nL min^−1^ for 9 min at 1% B and decreased to 300 µL min^−1^ in 1 min at 1% B. A gradient at a flowrate of 300 nL min^−1^ from 1 to 30% B from 10 to 85 min was applied, followed by an increase from 30 to 60% B in 15 min. Ninety-nine percent B was held for 10 min for flushing, and afterwards, flowrate was switched from 300 to 750 nL min^−1^ at 99% in 5 min followed by equilibration at 1% B for 35 min. The column and mass spectrometer were hyphenated using the Thermo Scientific^TM^ Nanospray Flex™ Ion Source (Thermo Fisher Scientific, San Jose, CA, USA) with the following parameter settings applied: spray voltage 1.5 kV, sheath and auxiliary gas 0, in-source CID 0, capillary temperature 250 °C, and S-Lens RF level 60. For MS1, the mass range for detection was set to 400–2000 *m/z* with a resolution setting of 70,000 at *m/z* 200, microscan 1, AGC target 3e6 with a maximum IT of 100 ms. For MS/MS, *m/z* range was set at 200–2000 with a resolution setting of 17,500 at *m/z* 200, AGC target value was 1e5, maximum injection time 50 ms, normalized collision energy (NCE) was 28, loop count 5, dynamic exclusion 10.0 ms, and microscan setting of 1.

### 4.5. Glycoprofiling of Released N-Glycans of MabThera^®^ and Reditux™ by xCGE-LIF

Analyses of released N-glycans were conducted on a xCGE-LIF-based glyXboxCE™ system (glyXera, Magdeburg, Germany), according to Cajic et al. [56]. For migration time alignment, crucial for glycan peak annotation via migration time matching with database entries of glyXbaseCE™ (glyXera), 1.0 µL of sample was mixed with 1.0 µL of 2nd NormMiX™ (glyXera) and 1.0 µL prediluted GeneScan™ 500 LIZ^®^ Size Standard. The mixture was combined with 6.0 µL glyXinject (glyXera) and subjected to xCGE-LIF analysis. The xCGE-LIF measurement samples were electrokinetically injected and analyzed with a running voltage of 15 kV for 40 min. Generated glycan data were analyzed with the glycoanalysis software glyXtoolCE™ (glyXera), automatically performing migration time alignment, raw data smoothing, peak picking, relative quantification, and peak/structure annotation.

### 4.6. Data Evaluation

Deconvolution of raw to zero-charge spectra was accomplished using the ReSpect™ algorithm with the sliding window deconvolution feature [26] of Thermo Scientific™ BioPharma Finder™ software version 3.0 (Thermo Fisher Scientific, San Jose, CA, USA). Deconvolution settings are reported in Table S8. 

Fractional abundances of MabThera^®^ and Reditux™ intact glycoforms were calculated using our in-house software fragquaxi v0.1 by semi-quantitation via extracted ion current chromatograms (EICCs) of MS1 ions. 

Peptide identification based on MS/MS fragment ion spectra was performed using Byonic™ v3.10.10 (Protein Metrics Inc., Cupertino, CA, USA). Parameters were set as follows: cleavage site(s) RK, Cleavage side C-terminal, Digestion specificity Fully specific, Missed cleavages 3, Precursor mass tolerance 10 ppm, Fragmentation type CID low energy, Fragment mass tolerance 20 ppm. Modifications were customized as follows: carbamidomethyl C fixed, Ammonia loss N rare 1, Deamidated N common 1, Dehydrated D, S, T, Y rare 1, Gln→pyro-Glu NTerm Q common 1, Oxidation M, W rare 1, Lys-loss Protein CTerm K common 1, and Hex K common1. The list of *N*-glycans added for identification is reported in Table S1, and the modification was set at rare1. Total common and Total rare max modification were both set at 2. 

Quantitation of peptide based on EICCs of MS1 ions in peptide mapping data was carried out using Skyline v20.2.0.343 (MacCoss Lab, Department of Genome Science, University of Washington). A list of peptides with the corresponding PTMs and retention times identified using Byonic™ was added in Skyline software, and peak identification and integration were validated manually. For glycation, peptides with missed cleavage were also considered. Resolution was set at 70,000 at 200 *m/z*, Orbitrap was selected as Precursor mass analyzer, Min % of base peak at 5%, *m/z* range from 400 to 2000 and Precursor charges from 1 to 5 and ion type p (precursor).

### 4.7. Data and Code Availability

Fragquaxi v0.1 is freely available on https://github.com/cdl-biosimilars/fragquaxi, accessed on 17 August 2021.

Raw files are available on Zenodo (DOI:10.5281/zenodo.5005513). 

## Data Availability

The Raw files of the data presented in this study are openly available in Zenodo at DOI:10.5281/zenodo.5005513.

## Supplementary Materials

The following are available online at https://www.mdpi.com/article/10.3390/ijms22169072/s1. Figure S1: RP-HPLC-MS analysis of reduced Reditux™; Figure S2: nano-HPLC-MS/MS peptide analyses of forced deamidated and non-stressed MabThera^®^ and Reditux™; Figure S3: nano-HPLC-MS/MS peptide analyses of forced glycated and non-stressedMabThera^®^ and Reditux™; Figure S4: Direct MS infusion of MabThera^®^ under native vs. denaturing condition; Figure S5: nano-HPLC-MS/MS glycopeptide analyses of MabThera^®^ and Reditux™; Table S1: Released glycan name, structure, and composition of MabThera^®^ and Reditux™; Table S2: Glycoform annotation of MabThera^®^; Table S3: Glycoform annotation of MabThera^®^ after carboxypeptidase B digestion; Table S4: Glycoform annotation of Reditux™; Table S5: Glycoform annotation of Reditux™ after carboxypeptidase B digestion; Table S6: Basic variant annotation of Reditux™ after carboxypeptidase B digestion; Table S7: Semi-quantitation of MabThera^®^ and Reditux™ glycoforms via extracted ion current chromatogram integration; Table S8: BioPharma Finder™ optimized deconvolution parameters.
