# Supplementary material for: Simultaneous Monitoring of Monoclonal Antibody Variants by Strong Cation-Exchange Chromatography Hyphenated to Mass Spectrometry to Assess Quality Attributes of Rituximab-Based Biotherapeutics
Source: Int J Mol Sci. 2021 Aug 23;22(16):9072. doi: 10.3390/ijms22169072 (PMC8396523; doi:10.3390/ijms22169072)
Supplement: Supplementary file 1 [file ijms-22-09072-s001.zip › ijms-1293176-supplementary.pdf]

Simultaneous monitoring of monoclonal antibody variants by strong cation-exchange chromatography hyphenated to mass spectrometry to assess critical attributes of rituximab-based biotherapeutics

Fiammetta Di Marco [1,2], Thomas Berger [1,2], Wolfgang Esser-Skala[1,2,3], Erdmann Rapp[4,5], Christof Regl[1,2] and Christian Huber [1,2,*]

[1] Department of Biosciences, Bioanalytical Research Labs, University of Salzburg, Hellbrunner Straße 34, 5020 Salzburg, Austria
[2] Christian Doppler Laboratory for Innovative Tools for Biosimilar Characterization, University of Salzburg, Hellbrunner Straße 34, 5020 Salzburg, Austria
[3] Department of Biosciences, Computational Systems Biology Group, University of Salzburg, Hellbrunner Straße 34, 5020 Salzburg, Austria
[4] glyXera GmbH, Brenneckestraße 20 - ZENIT, 39120 Magdeburg, Germany
[5] Max Planck Institute for Dynamics of Complex Technical Systems, Sandtorstraße 1, 39106 Magdeburg, Germany
* Correspondence: c.huber@sbg.ac.at

Table of Contents

Supplementary Figures 3

Figure S1. RP-HPLC-MS analysis of reduced Reditux™ 3

Figure S2. nano-HPLC-MS/MS peptide analyses of forced deamidated and control MabThera® and Reditux™... 4

Figure S3. nano-HPLC-MS/MS peptide analyses of forced glycated and control MabThera® and Reditux™........ 5

Figure S4. Direct MS infusion of MabThera® under native vs denaturing condition........ 6

Figure S5. nano-HPLC-MS/MS glycopeptide analyses of MabThera® and Reditux™ 7

Supplementary Tables 8

Table S1. Released glycan name, structure and composition of MabThera® and Reditux™ 8

Table S2. Glycoform annotation of MabThera®........ *9*

Table S3. Glycoform annotation of MabThera® after carboxypeptidase B digestion........ 10

Table S4. Glycoform annotation of Reditux™ 11

Table S5. Glycoform annotation of Reditux™ after carboxypeptidase B digestion 12

Table S6. Basic variant annotation of Reditux™ after carboxypeptidase B digestion 13

Table S7. Semi-quantitation of MabThera® and Reditux™ glycoforms via extracted ion current chromatogram integration 14

Table S8. BioPharma Finder™ 3.0 optimized deconvolution parameters 15

Supplementary Figures

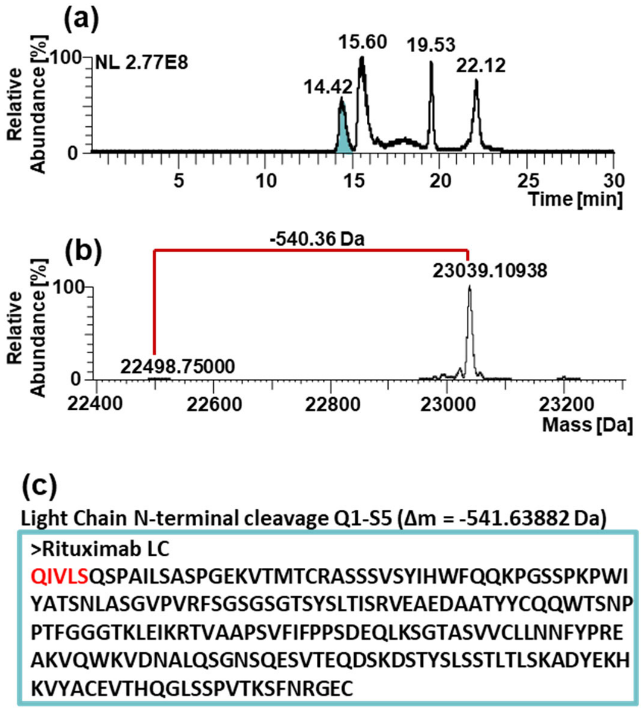

Figure S1. (a) Chromatogram of reduced Reditux™ (5 mM TCEP) obtained by RP-HPLC-MS analysis. Heavy and light chains are dissociated due to the reducing condition and separated by RP-HPLC. **(b)** Deconvoluted mass spectrum of the light chain corresponding to chromatographic peak at 14.42 min (highlighted in light blue). A variant differing in mass by -540.36 Da (22498.75000 Da) can be observed in the spectrum. (**c**) Rituximab light chain sequence. The variant at 22498.75000 Da could arise from cleavage of the first five residues (Q1-S5, indicated in red) of the light chain N-terminus resulting in a Δm of -541.63882 Da.

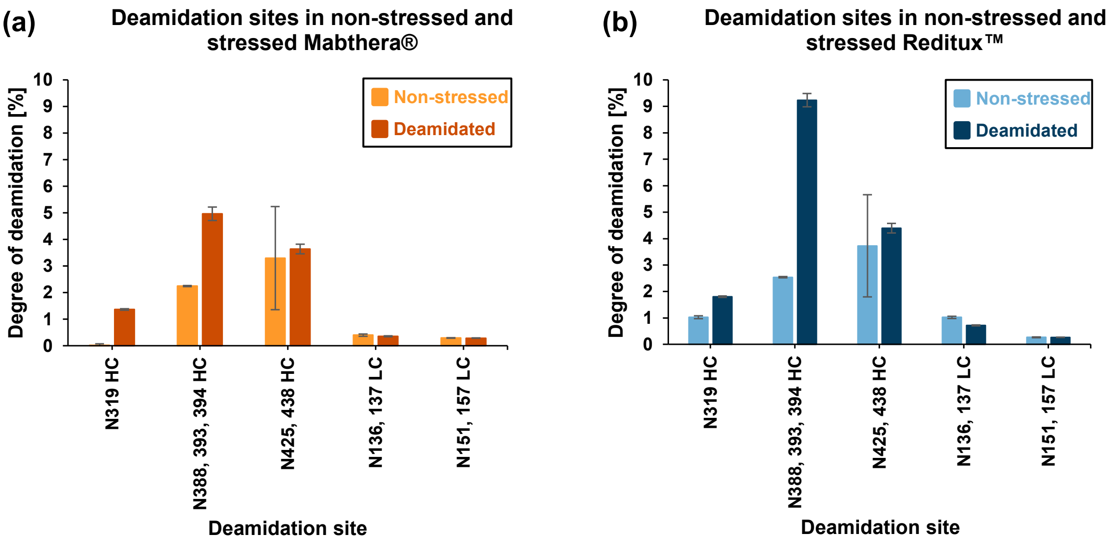

(c)

>Rituximab HC
QVQLQQPGAELVKPGASVKMSCKASGYTFTSYNMHWVKQTPGRGLEWIGAIYPGNGDTSYNQKFKGKATLTADKSSSTAYMQLSSLTSEDSAVYYCARSTYYGGDWY
FNVWGAGTTVTVSAASTKGPSVFPLAPSSKSTSGGTAALGCLVKDYFPEPVTVSWNSGALTSGVHTFPAVLQSSGLYSLSSVVTVPSSSLGTQTYICNVNHKPSNTKVDKK
AEPKSCDKTHTCPPCPAPELLGGPSVFLFPPKPKDTLMISRTPEVTCVVVDVSHEDPEVKFNWYVDGVEVHNAKTKPREEQYNSTYRVVSVLTVLHQDWLN$_{319}$GKEYKCK
VSNKALPAPIEKTISKAKGQPREPQVYTLPPSRDELTKNQVSLTCLVKGFYPSDIAVEWESN$_{388}$GQPEN$_{393}$N$_{394}$YKTTPPVLDSDGSFFLYSKLTVDKSRWQQGN$_{425}$VFSCS
VMHEALHN$_{438}$HYTQKSLSLSPGK
>Rituximab LC
QIVLSQSPAILSASPGEKVTMTCRASSSVSYIHWFQQKPGSSPKPWIYATSNLASGVPVRFSGSGSGTSYSLTISRVEAEDAATYYCQQWTSNPPTFGGGTKLEIKRTVAAP
SVFIFPPSDEQLKSGTASVVCLLN$_{136}$N$_{137}$FYPREAKVQWKVDN$_{151}$ALQSGN$_{157}$SQESVTEQDSKDSTYSLSSTLTLSKADYEKHKVYACEVTHQGLSSPVTKSFNRGEC

Figure S2. Bar charts of peptide deamidation degree in **(a)** control and forced deamidated MabThera®, **(b)** non-stressed and forced deamidated Reditux™. Deamidation sites are indicated with residue position and the corresponding subunits (Heavy Chain, HC or Light Chain, LC) in the x-axis. Quantitation of peptide was based on peak integration of EICCs of MS1 ions in peptide mapping data, thus asparagine residues are considered together when belonging to the same peptide. **(c)** Rituximab amino acid sequence with deamidation sites reported in red.

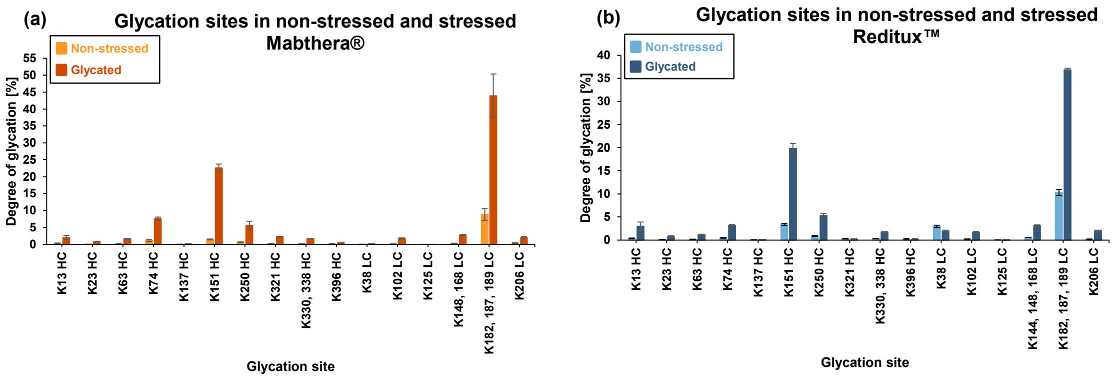

(c)

>Rituximab HC

QVQLQQPGAELVK_{13}PGASVKMSCK_{23}ASGYTFTSYNMHWVKQTPGRGLEWIGAIYPGNGDTSYNQK_{63}FKGKATLTADK_{74}SSSTAYMQLSSLTSEDSAVYYCARSTYYGGDWYFNVWGA
GTTVTVSAASTKGPSVFPLAPSSK_{137}STSGGTAALGCLVK_{151}DYFPEPVTVSWNSGALTSGVHTFPAVLQSSGLYSLSSVVTVPSSSLGTQTYICNVNHKPSNTKVDKKAEPKSCDKTHTCPPC
PAPELLGGPSVFLFPPK_{250}PKDTLMISRTPEVTCVVVDVSHEDPEVKFNWYVDGVEVHNAKTKPREEQYNSTYRVVSVLTVLHQDWLNGK_{321}EYKCKVSNK_{330}ALPAPIEK_{338}TISKAKGQ
PREPQVYTLPPSRDELTKNQVSLTCLVKGFYPSDIAVEWESNGQPENNYK_{396}TTPPVLDSDGSFFLYSKLTVDKSRWQQGNVFSCSVMHEALHNHYTQKSLSLSPGK

>Rituximab LC

QIVLSQSPAILSASPGEKVTMTCRASSSVSYIHWFQQK_{38}PGSSPKPWIYATSNLASGVPVRFSGSGSGTSYSLTISRVEAEDAATYYCQQWTSNPPTFGGGTK_{102}LEIKRTVAAPSVFIFPPSD
EQLK_{125}SGTASVVCLLNNFYPREAK_{144}VQWK_{148}VDNALQSGNSQESVTEQDSK_{168}DSTYSLSSTLTLSK_{182}ADYEK_{187}HK_{189}VYACEVTHQGLSSPVTK_{206}FNRGEC

Figure S3. Bar charts of peptide glycation degree in **(a)** control and forced glycated MabThera®, **(b)** non-stressed and forced glycated Reditux™. Glycation sites are indicated with residue position and the corresponding subunits (Heavy Chain, HC or Light Chain, LC) in the x-axis. Quantitation of peptide was based on peak integration of EICCs of MS1 ions in peptide mapping data, thus lysine residues are considered together when belonging to the same peptide. **(c)** Rituximab amino acid sequence with glycation sites reported in red.

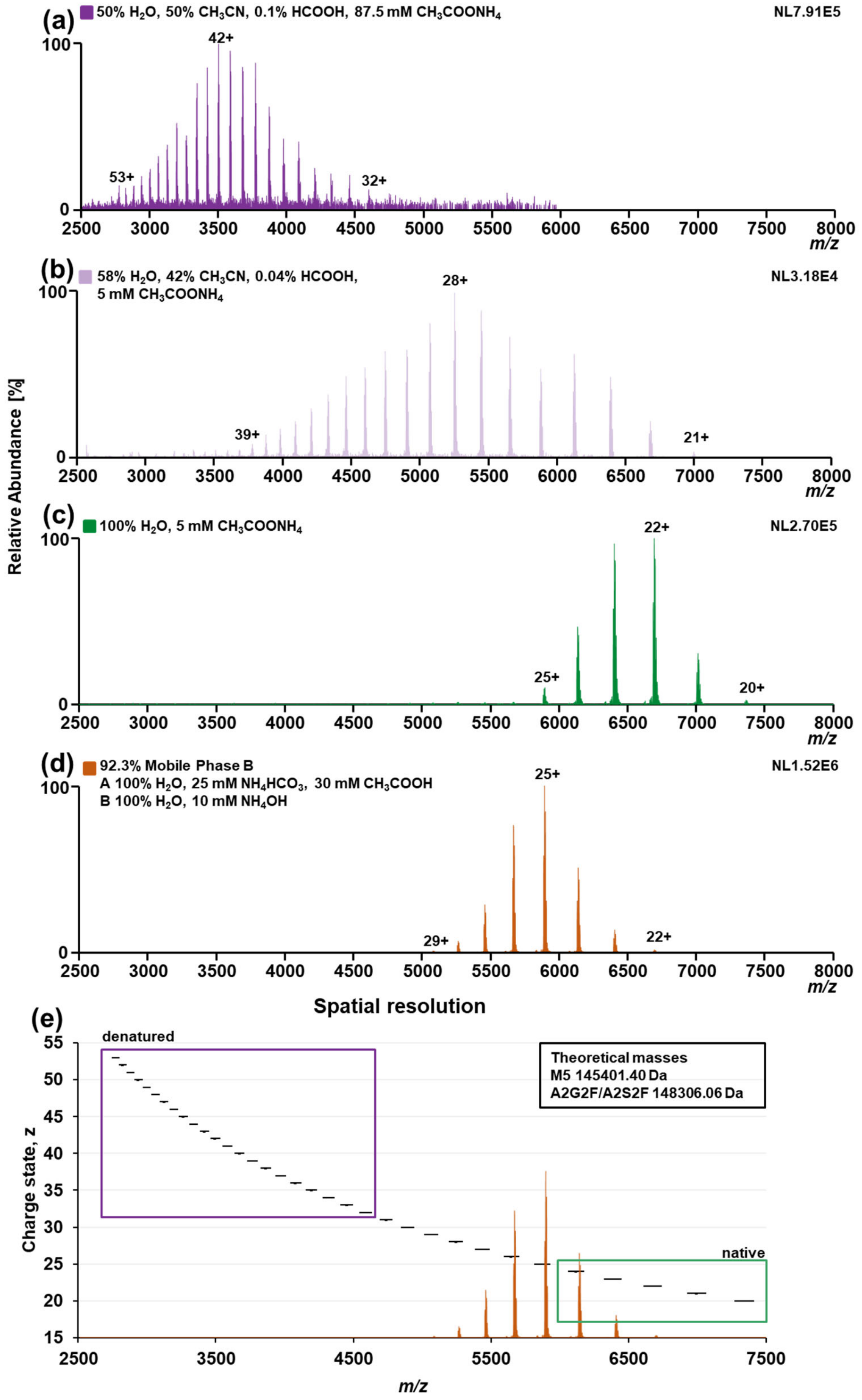

Figure S4. Direct infusion MS analyses of MabThera® in **(a)** a denaturing solution, **(b)** a partially denaturing solution,**(c)** a solution maintaining the mAb in the quasi-native state. **(d)** SCX-HPLC-MS analysis of MabThera® eluting at 92.3% Mobile Phase B (A: 100% H_2O, 25 mM NH_4HCO_3, 30 mM CH_3COOH, B: 100% H_2O, 10 mM NH_4OH). **(e)** Charge state dependent spatial resolution plot. Charge envelope range were calculated considering the theoretical masses of the first (M5, 145401.40 Da) and the last (A2G2F/A2S2F, 148306.06 Da) glycoform observed. The charge envelope partially overlaps going towards higher charge state (denaturing condition). The mass spectrum of MabThera® acquired by SCX-HPLC-MS analysis (orange) is in quasi- native charge range.

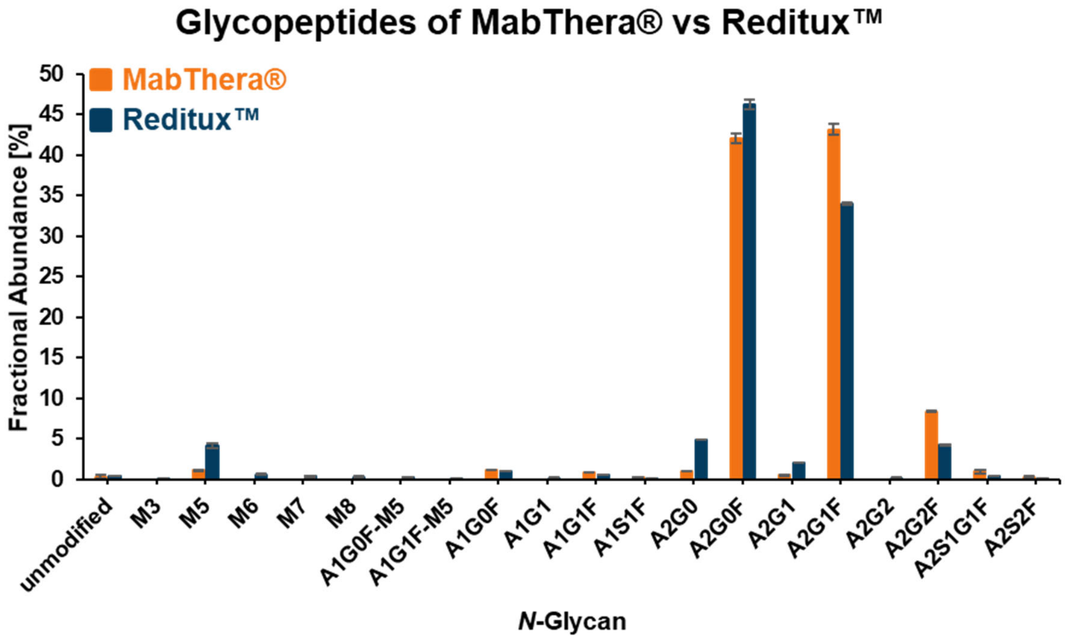

Figure S5. Bar chart reporting *N*-glycans fractional abundances obtained by nano-RPLC-MS/MS of glycopeptides of MabThera® (orange) and Reditux™ (blue). MabThera® showed a higher degree of sialylation and galactosylation compared to Reditux™ that exhibited instead a higher abundance of oligomannose and afucosylated variants. Glycan structures, names and compositions are collected in Table S1.

Supplementary Tables

Table S1. Name, structure and monosaccharide composition of MabThera® and Reditux™ *N*-glycans elucidated by xCGE-LIF. A green cell indicates presence, a red cell absence of that glycan in the drug product.

N-Glycans				
**Name**	**Structure**	**Monosaccharide composition**	**MabThera®**	**Reditux™**
**M3**	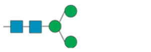	HexNac(2)Hex(3)Neu5Ac(0)Fuc(0)		
**M5**	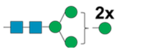	HexNac(2)Hex(5)Neu5Ac(0)Fuc(0)		
**M6**	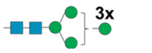	HexNac(2)Hex(6)Neu5Ac(0)Fuc(0)		
**M7**	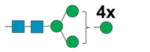	HexNac(2)Hex(7)Neu5Ac(0)Fuc(0)		
**M8**	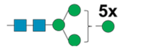	HexNac(2)Hex(8)Neu5Ac(0)Fuc(0)		
**A1G0F-M5**	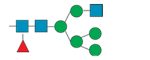	HexNac(3)Hex(5)Neu5Ac(0)Fuc(1)		
**A1G1F-M5**	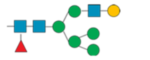	HexNac(3)Hex(6)Neu5Ac(0)Fuc(1)		
**A1G0F**	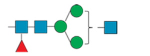	HexNac(3)Hex(3)Neu5Ac(0)Fuc(0)		
**A1G1**	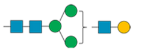	HexNac(3)Hex(4)Neu5Ac(0)Fuc(0)		
**A1G1F**	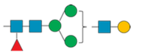	HexNac(3)Hex(4)Neu5Ac(0)Fuc(1)		
**A1S1F**	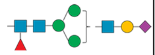	HexNac(3)Hex(4)Neu5Ac(1)Fuc(1)		
**A2G0**	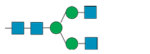	HexNac(4)Hex(3)Neu5Ac(0)Fuc(0)		
**A2G0F**	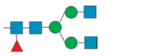	HexNac(4)Hex(3)Neu5Ac(0)Fuc(1)		
**A2G1**	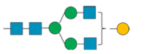	HexNac(4)Hex(4)Neu5Ac(0)Fuc(0)		
**A2G1F**	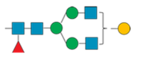	HexNac(4)Hex(4)Neu5Ac(0)Fuc(1)		
**A2G2**	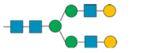	HexNac(4)Hex(5)Neu5Ac(0)Fuc(0)		
**A2G2F**	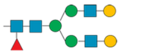	HexNac(4)Hex(5)Neu5Ac(0)Fuc(1)		
**A2S1G1F**	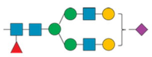	HexNac(4)Hex(5)Neu5Ac(1)Fuc(1)		
**A2S2F**	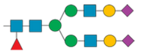	HexNac(4)Hex(5)Neu5Ac(2)Fuc(1)		

Table S2. Glycoform annotation of the deconvoluted mass spectrum of MabThera® (Figure 1b and 4) reporting variant name, experimental mass, theoretical mass, mass deviation in ppm, fractional abundance, retention time range and retention time apex. Theoretical masses were calculated considering N-terminal pyro-Glu as a fixed modification. Glycan structures, names and compositions are collected in Table S1.

MabThera® annotation						
**Variant Name**	**Experimental Mass [Da]**	**Theoretical Mass [Da]**	**Mass deviation [ppm]**	**Fractional Abundance [%]**	**RT range [min]**	**RT apex [min]**
M5_0K	145411.05	145401.40	66.3	0.01	9.98 - 12.03	11.52
A2G0F_0K	145631.87	145629.65	15.3	0.19	9.24 - 12.03	11.52
A2G1F_0K	145792.49	145791.79	4.8	0.22	8.98 - 12.03	10.73
A2G2F_0K	145953.88	145953.93	-0.3	0.09	8.98 - 12.03	11.52
unidentified	146252.17			0.00	9.50 - 11.24	10.49
unidentified	146508.84			0.01	9.98 - 11.50	11.00
M5/M5_0K	146623.72	146618.49	35.6	0.40	9.77 - 12.03	11.52
A2G0/M5_0K	146703.51	146700.60	19.8	0.02	9.24 - 12.03	11.52
A2G1/M5_0K	146858.12	146862.74	-31.4	0.09	8.98 - 12.03	11.52
A2G0/A2G0F_0K	146926.13	146928.84	-18.5	0.08	8.98 - 12.03	11.52
A2G0F/A2G0F_0K	147076.43	147074.99	9.9	19.02	8.98 - 12.03	11.52
A2G0F/A2G1F_0K	147236.40	147237.13	-4.9	32.85	8.98 - 12.03	11.52
A2G1F/A2G1F_0K or A2G0F/A2G2F_0K	147397.94	147399.27	-9.0	28.85	8.98 - 12.03	11.26
A2G1F/A2G2F_0K	147559.97	147561.41	-9.7	14.16	8.98 - 12.03	11.26
A2G2F/A2G2F_0K	147720.06	147723.55	-23.6	1.81	8.98 - 12.03	11.26
A2G1F/A2S1G1F_0K	147855.90	147852.66	21.9	0.75	8.98 - 12.03	10.01
A2G2F/A2S1G1F_0K	148012.41	148014.80	-16.2	0.19	9.50 - 11.77	10.26
A2G1F/A2S2F_0K	148146.29	148143.92	16.0	0.37	8.98 - 12.03	9.48
A2G2F/A2S2F_0K	148306.06	148306.06	0.0	0.17	8.98 - 11.00	9.48
unidentified	148474.93			0.01	8.98 - 10.76	9.48
unidentified	148681.42			0.01	9.77 - 11.77	10.73
unidentified	148842.84			0.01	9.77 - 11.24	10.49
unidentified	148944.16			0.00	9.50 - 12.03	11.52

Table S3. Glycoform annotation of the deconvoluted mass spectrum of MabThera® after carboxypeptidase B (CpB) treatment (Figure 1b) reporting variant name, experimental mass, theoretical mass, mass deviation in ppm, fractional abundance, retention time range and retention time apex. Theoretical masses were calculated considering N-terminal pyro-Glu as a fixed modification. Glycan structures, names and compositions are collected in Table S1.

MabThera® CpB annotation

Variant Name	Experimental Mass [Da]	Theoretical Mass [Da]	Mass deviation [ppm]	Fractional Abundance [%]	RT range [min]	RT apex [min]
A2G0F_0K	145631.55	145629.65	13.0	0.17	10.21 - 12.01	11.76
A2G1F_0K	145793.74	145791.79	13.4	0.35	9.21 - 12.01	11.76
A2G2F_0K	145955.01	145953.93	7.4	0.11	10.21 - 12.01	11.76
unidentified	146506.88			0.02	11.16 - 12.01	11.76
M5/M5_0K	146623.46	146618.49	33.9	0.21	10.32 - 12.01	11.76
A2G1/M5_0K	146856.08	146862.74	-45.4	0.13	9.37 - 12.01	11.76
A2G0/A2G0F_0K	146926.51	146928.84	-15.9	0.23	9.37 - 12.01	11.76
A2G0F/A2G0F_0K	147076.55	147074.99	10.7	18.11	9.00 - 12.01	11.76
A2G0F/A2G1F_0K	147236.80	147237.13	-2.2	31.67	9.00 - 12.01	11.76
A2G1F/A2G1F_0K or A2G0F/A2G2F_0K	147398.38	147399.27	-6.0	28.75	9.00 - 12.01	11.76
A2G1F/A2G2F_0K	147561.21	147561.41	-1.3	13.53	9.00 - 12.01	11.76
A2G2F/A2G2F_0K	147722.43	147723.55	-7.6	1.61	9.00 - 12.01	11.76
A2G1F/A2S1G1F_0K	147856.73	147852.66	27.5	1.56	9.21 - 11.75	11.15
A2G2F/A2S1G1F_0K	148014.33	148014.80	-3.2	0.42	9.11 - 12.01	11.15
A2G1F/A2S2F_0K	148147.29	148143.92	22.7	0.74	9.00 - 11.88	10.55
A2G2F/A2S2F_0K	148308.14	148306.06	14.0	0.41	9.11 - 11.66	10.56

Table S4. Glycoform annotation of the deconvoluted mass spectra of Reditux™ (Figure 1c-e) reporting variant name, experimental mass, theoretical mass, mass deviation in ppm, fractional abundance, retention time range and retention time apex. Theoretical masses were calculated considering N-terminal pyro-Glu as a fixed modification. Glycan structures, names and compositions are collected in Table S1.

Reditux™ annotation

Variant Name	Experimental Mass [Da]	Theoretical Mass [Da]	Mass deviation [ppm]	Fractional Abundance [%]	RT range [min]	RT apex [min]
M5_0K	145410.20	145401.40	60.5	0.08	11.50 - 12.47	12.32
A2G0F_0K	145631.75	145629.65	14.4	0.19	11.50 - 12.47	12.01
A2G1F_0K	145790.19	145791.79	-11.0	0.20	11.50 - 12.47	12.01
A2G2F_0K	145948.23	145953.93	-39.0	0.03	11.50 - 12.03	11.64
A2S1G0F_0K	146094.50	146083.05	78.4	0.11	11.50 - 12.47	12.32
unidentified	146254.66			0.00	11.50 - 12.26	11.64
M5/M5_0K	146620.97	146618.49	16.9	3.87	11.50 - 12.47	12.1
A2G0/A2G0_0K	146790.01	146782.70	49.8	0.53	11.50 - 12.32	11.8
A2G0/A2G0F_0K	146924.44	146928.84	-29.9	3.63	11.50 - 12.47	11.88
A2G0F/A2G0F_0K	147077.94	147074.99	20.1	29.80	11.50 - 12.47	11.95
A2G0F/A2G1F_0K	147239.24	147237.13	14.4	34.70	11.50 - 12.47	11.88
A2G1F/A2G1F_0K or A2G0F/A2G2F_0K	147400.26	147399.27	6.7	21.63	11.50 - 12.47	11.88
A2G1F/A2G2F_0K	147560.25	147561.41	-7.8	3.61	11.50 - 12.16	11.71
A2G2F/A2G2F_0K	147708.05	147723.55	-104.9	0.49	11.50 - 12.16	11.64
A2G1F/A2S1G1F_0K	147856.13	147852.66	23.4	0.08	11.50 - 11.94	11.64
unidentified	147984.13			0.11	11.94 - 12.47	12.32
A2G1F/A2S2F_0K	148153.43	148143.92	64.2	0.05	11.87 - 12.42	12.17
A2G2F/A2S2F_0K	148304.16	148306.06	-12.8	0.02	11.94 - 12.42	12.17
unidentified	148450.58			0.03	11.79 - 12.32	12.01
A2S1G1F/A2S2F_0K	148588.03			0.01	11.50 - 12.47	11.64
M5_1K	145538.00	145529.58	57.9	0.01	12.84 - 13.34	13.20
A2G0F_1K	145761.47	145757.82	25.0	0.16	12.68 - 13.34	13.20
A2G1F_1K	145921.64	145919.96	11.5	0.15	12.58 - 13.34	13.20
A2G2F_1K	146081.62	146082.10	-3.3	0.07	12.74 - 13.34	13.20
unidentified	146234.50			0.08	12.58 - 13.34	13.20
unidentified	146384.86			0.10	12.58 - 13.34	13.11
unidentified	146539.10			1.08	12.58 - 13.34	13.20
unidentified	146700.72			0.27	12.68 - 13.19	12.83
unidentified	146864.86			0.13	12.58 - 13.12	12.89
A2G0/A2G0F_1K	147055.78	147057.02	-8.4	3.22	12.74 - 13.34	13.20
A2G0F/A2G0F_1K	147206.07	147203.16	19.8	28.55	12.68 - 13.34	13.20
A2G0F/A2G1F_1K	147367.02	147365.30	11.7	39.42	12.58 - 13.34	13.20
A2G1F/A2G1F_1K or A2G0F/A2G2F_1K	147529.70	147527.44	15.3	21.79	12.58 - 13.34	13.20
A2G1F/A2G2F_1K	147694.70	147689.58	34.7	4.43	12.58 - 13.34	13.20
A2G2F/A2G2F_1K	147851.95	147851.72	1.5	0.25	12.58 - 13.34	12.73
A2G1F/A2S1G1F_1K	147995.66	147980.84	100.2	0.03	12.68 - 13.26	12.83
A2G2F/A2S1G1F_1K	148133.03	148142.98	-67.1	0.04	12.74 - 13.19	12.98
unidentified	148252.85			0.06	12.89 - 13.34	13.20
unidentified	148407.37			0.12	12.58 - 13.34	13.20
unidentified	148572.29			0.04	12.84 - 13.34	13.20
unidentified	145739.58			0.02	14.49 - 15.15	14.96
A2G0F_2K	145888.38	145886.00	16.4	0.17	14.23 - 15.15	14.77
A2G1F_2K	146048.16	146048.14	0.1	0.12	14.49 - 15.15	14.96
A2G2F_2K	146203.22	146210.28	-48.3	0.02	14.59 - 15.15	14.96
unidentified	146353.75			0.06	14.38 - 15.15	14.96
unidentified	146524.81			0.09	14.23 -15.15	14.41
unidentified	146670.63			0.25	14.23 - 15.05	14.41
M5/M5_2K	146877.43	146874.84	17.6	0.39	14.59 - 15.15	14.96
A2G0/A2G0_2K	147038.77	147039.05	-1.9	0.09	14.49 - 15.05	14.77
A2G0/A2G0F_2K	147186.12	147185.19	6.3	3.98	14.28 - 15.15	14.96
A2G0F/A2G0F_2K	147334.13	147331.33	19.0	32.55	14.23 - 15.15	14.96
A2G0F/A2G1F_2K	147495.66	147493.47	14.8	38.86	14.23 - 15.15	14.87
A2G1F/A2G1F_2K or A2G0F/A2G2F_2K	147656.06	147655.61	3.0	19.50	14.23 - 15.15	14.87
A2G1F/A2G2F_2K	147821.64	147817.75	26.3	3.69	14.23 - 15.15	14.77
A2G2F/A2G2F_2K	147990.79	147979.89	73.6	0.11	14.28 - 15.15	14.96

Table S5. Glycoform annotation the deconvoluted mass spectrum of Reditux™ after carboxypeptidase B (CpB) treatment (Figure 1h and Figure 4) reporting variant name, experimental mass, theoretical mass, mass deviation in ppm, fractional abundance, retention time range and retention time apex. Theoretical masses were calculated considering N-terminal pyro-Glu as a fixed modification. Glycan structures, names and compositions are collected in Table S1.

Reditux™ CpB annotation

Variant Name	Experimental Mass [Da]	Theoretical Mass [Da]	Mass deviation [ppm]	Fractional Abundance [%]	RT range [min]	RT apex [min]
M5_0K	145404.26	145401.40	19.6	0.02	10.55 - 11.96	11.61
A2G0F_0K	145633.35	145629.65	25.4	0.17	10.18 - 11.96	11.61
A2G1F_0K	145793.11	145791.79	9.1	0.15	9.66 - 11.96	11.61
A2G2F_0K	145951.96	145953.93	-13.5	0.02	9.50 - 11.60	11.06
A2S1G0F_0K	146095.07	146083.05	82.3	0.04	10.71 - 11.96	11.61
unidentified	146259.78			0.01	10.39 - 11.96	11.61
M5/M5_0K	146620.20	146618.49	11.6	1.60	9.50 - 11.96	11.61
A2G0/A2G0_0K	146783.34	146782.70	4.3	0.14	9.50 - 11.41	10.90
A2G0/A2G0F_0K	146929.03	146928.84	1.2	3.71	9.50 - 11.96	11.61
A2G0F/A2G0F_0K	147077.87	147074.99	19.6	26.82	9.50 - 11.96	11.61
A2G0F/A2G1F_0K	147237.46	147237.13	2.3	35.28	9.50 - 11.96	11.61
A2G1F/A2G1F_0K or A2G0F/A2G2F_0K	147398.63	147399.27	-4.3	23.02	9.66 - 11.96	11.61
A2G1F/A2G2F_0K	147561.06	147561.41	-2.3	6.92	9.50 - 11.96	11.61
A2G2F/A2G2F_0K	147715.68	147723.55	-53.3	0.61	10.39 - 11.60	11.06
A2G1F/A2S1G1F_0K	147857.54	147852.66	33.0	0.31	10.18 - 11.60	10.90
A2G2F/A2S1G1F_0K	148003.91	148014.80	-73.6	0.12	9.50 - 11.07	10.36
A2G1F/A2S2F_0K	148150.60	148143.92	45.1	0.15	9.66 - 11.96	10.55
A2G2F/A2S2F_0K	148310.86	148306.06	32.4	0.05	9.50 - 11.25	10.55
unidentified	148843.50			0.02	9.87 - 11.96	11.61

Table S6. Basic variant annotation of deconvoluted mass spectrum of Reditux™ after carboxypeptidase B (CpB) treatment (Figure 1i) reporting variant name, experimental mass, theoretical mass, mass deviation in ppm, fractional abundance, retention time range and retention time apex. The basic variant arising from N-terminal cleavage of residues Q1-S5 of the light chain (Figure S1) is indicated as (LC pyro-Glu (Q6) - N-term Q1-S5). Unidentified basic PTMs could be attributed to some modifications with a basic nature not-resolvable by MS at intact level, e.g C-terminal proline amidation, succinimide formation, methionine oxidation, glutamic acid instead of pyro-glutamate, isomerization of aspartate, disulfide shuffling. Glycan structures, names and compositions are collected in Table S1.

Reditux™ CpB basic variant annotation, RT range (12.00-17.00 min)

Variant Name	Experimental Mass [Da]	Theoretical Mass [Da]	Mass deviation [ppm]	Fractional Abundance [%]	RT range [min]	RT apex [min]
Unidentified basic PTM	145300.43			0.03	14.02 - 15.39	14.68
Unidentified basic PTM + M5_0K	145404.36			0.08	11.99 - 14.23	12.46
Unidentified basic PTM + A2G0F_0K	145636.57			0.24	11.99 - 14.50	12.46
Unidentified basic PTM + A2G1F_0K	145791.17			0.18	11.99 - 14.50	12.46
M5/M5_0K (LC pyro-Glu (Q6) - N-term Q1-S5)	146094.49	146076.86	120.7	0.23	11.99 - 14.23	12.46
A2G0/A2G0F_0K (LC pyro-Glu (Q6) - N-term Q1-S5)	146390.07	146387.21	19.6	0.53	12.24 - 14.23	13.10
A2G0F/A2G0F_0K (LC pyro-Glu (Q6) - N-term Q1-S5)	146538.93	146533.35	38.1	3.75	12.45 - 14.71	13.37
A2G0F/A2G1F_0K (LC pyro-Glu (Q6) - N-term Q1-S5)	146698.59	146695.49	21.1	4.09	12.24 - 14.92	13.37
A2G1F/A2G1F_0K (LC pyro-Glu (Q6) - N-term Q1-S5)	146859.13	146857.63	10.2	1.61	12.45 - 14.02	13.10
Unidentified basic PTM + A2G0/A2G0F_0K	146929.23			0.74	13.34 - 16.96	14.26
Unidentified basic PTM + A2G0F/A2G0F_0K	147080.44			27.51	11.99 - 16.96	12.46
Unidentified basic PTM + A2G0F/A2G1F_0K	147244.84			21.00	11.99 - 13.34	12.46
Unidentified basic PTM + A2G1F/A2G1F_0K or A2G0F/A2G2F_0K	147405.01			10.17	11.99 - 13.34	12.46
Unidentified basic PTM + A2G1F/A2G2F_0K	147564.80			3.81	11.99 - 16.96	12.46
Unidentified basic PTM + A2G2F/A2G2F_0K	147722.76			0.50	12.66 - 16.28	14.26
Unidentified basic PTM + A2G1F/A2S1G1F_0K	147855.66			0.09	12.24 - 14.92	12.69

Table S7. Results of fractional abundance semi-quantitation of MabThera® and Reditux™ glycoforms via Extracted ion current chromatograms (EICCs) integration using Fragquaxi. CpB indicates the treatment with carboxypeptidase. Bar charts referred to MabThera® vs Reditux™ CpB and Reditux™ 0K, 1K and 2K can be found in the main manuscript (Figure 5). Glycan structures, names and compositions are collected in Table S1.

	MabThera® CpB		Reditux™ CpB		MabThera®		Reditux™ 0K		Reditux™ 1K		Reditux™ 2K	
Glycoforms	Average [%]	STD [%]	Average [%]	STD [%]	Average [%]	STD [%]	Average [%]	STD [%]	Average [%]	STD [%]	Average [%]	STD [%]
M5	0.03	0.01	0.08	0.00	0.04	0.02	0.14	0.01	0.06	0.01	0.10	0.03
A2G0F	0.31	0.00	0.30	0.01	0.31	0.01	0.31	0.00	0.31	0.01	0.29	0.02
A2G1F	0.42	0.01	0.27	0.00	0.42	0.00	0.30	0.01	0.26	0.01	0.23	0.01
A2G2F	0.12	0.01	0.08	0.01	0.12	0.01	0.12	0.01	0.20	0.01	0.11	0.01
A2S1G0F	0.03	0.01	0.13	0.01	0.04	0.02	0.15	0.01	0.12	0.00	0.13	0.00
M5/M5	0.50	0.01	2.30	0.02	0.45	0.01	3.24	0.03	1.07	0.02	0.93	0.02
A2G0/M5	0.27	0.07	0.58	0.03	0.38	0.01	0.18	0.01	0.67	0.04	0.98	0.07
A2G0F/M5	0.57	0.04	0.86	0.03	0.64	0.04	0.82	0.01	0.43	0.02	1.08	0.11
A2G1/M5	0.62	0.04	0.88	0.03	0.70	0.04	0.78	0.01	0.39	0.02	0.64	0.06
A2G0/A2G0	0.01	0.00	0.80	0.01	0.02	0.01	1.06	0.02	0.55	0.10	0.81	0.04
A2G0F/A2G0	0.47	0.01	4.28	0.03	0.44	0.01	4.04	0.05	6.41	0.45	5.63	0.03
A2G0F/A2G0F	19.87	0.21	27.13	0.15	19.70	0.26	24.33	0.15	28.63	0.31	28.13	0.42
A2G0F/A2G1F	33.50	0.17	34.93	0.06	33.40	0.17	33.17	0.23	35.00	0.30	35.37	0.25
A2G1F/A2G1F	28.20	0.00	20.10	0.10	28.20	0.10	20.90	0.10	19.13	0.06	19.13	0.06
A2G1F/A2G2F	10.73	0.06	5.55	0.08	10.80	0.10	6.83	0.21	4.90	0.03	4.77	0.07
A2G2F/A2G2F	2.15	0.03	0.92	0.01	2.15	0.05	1.66	0.06	0.86	0.04	0.79	0.06
A2G1F/A2S1G1F	1.04	0.09	0.38	0.02	1.08	0.13	1.01	0.01	0.45	0.03	0.33	0.04
A2G1F/A2S2F	0.41	0.04	0.15	0.01	0.45	0.05	0.33	0.01	0.15	0.02	0.19	0.06
A2G2F/A2S1G1F	0.45	0.04	0.17	0.01	0.45	0.06	0.43	0.02	0.21	0.03	0.22	0.06
A2G2F/A2S2F	0.25	0.03	0.09	0.01	0.27	0.04	0.22	0.04	0.13	0.03	0.16	0.04

Table S8. Optimized deconvolution parameters used in BioPharma Finder™ 3.0 for the mass spectra of MabThera® and Reditux™ before and after carboxypeptidase treatment (CpB).

Deconvolution Parameters BioPharma Finder 3.0									
		Parameter	MabThera®	MabThera® CpB	Reditux™ 0K	Reditux™ 1K	Reditux™ 2K	Reditux™ CpB	Reditux™ CpB basic variants
Chromatogram and Source Spectra	Chromatogram Parameters	*m/z* range	2500-8000		5000-7000			2500-8000	
	Source spectra method		sliding window						
	Sliding Windows Definition	RT Range [mim]	9.00-12.00	9.00-12.00	11.50-12-50	12.60-13-40	14.20-15-20	9.50-12-00	12.00-17.00
		Target Avg Spectrum Width [min]	1.000	0.480	0.300	0.300	0.380	0.700	0.900
		Target Avg Spectrum % Offset	25						
	Sliding Windows Merging Parameters	Merge Tolerance [ppm]	20						
		Max RT Gap [min]	1						
		Min. Number of Detected Intervals	3						
Deconvolution Algorithm, basic	Deconvolution Results Filter	Output Mass Range	145000-149000						
		Deconvolution Spectra Display Mode	Isotopic Profile						
	Charge State Distribution	Deconvolution Mass Tolerance [ppm]	20						
	Choice of Peak Model	Choice of Peak Model	Intact Protein						
		Resolution at 400 *m/z*	Raw File Specific						
Deconvolution Algorithm, advanced	Deconvolution Algorithm		ReSpect™						
	Charge State Distribution	Model Mass Range	145000-149000						
		Charge State Range	20-28		23-27				
		Minimum Adjacent Charges (low and high model mass)	2 to 2						
	Noise Parameters	Rel. Abundance Threshold (%)	0.00						
	Deconvolution Quality	Quality Score Threshold	0.00						
	Choice of Peak Model	Target Mass	147000						
	Peak Model Parameters	Number of Peak Models	1						
		Left/Right Peak Shape	Left 2.00, Right 2.00					Left 2.00, Right 3.00	Left 2.00, Right 2.00
	Peak Filter Parameters	Peak Detection Minimum Significance Measure	1.00		1.50			1.00	
		Peak Detection Quality Measure [%]	95						
	Specialized Parameters	Peak Model Width Factor	1.00		1.80			1.50	
		Intensity Threshold Scale	0.00001						
	Deconvolution Parameters	Noise Compesation	✓						
		Charge Carrier	H+						
